# Nutraceutical Characterization of Anthocyanin-Rich Fruits Produced by “Sun Black” Tomato Line

**DOI:** 10.3389/fnut.2019.00133

**Published:** 2019-08-28

**Authors:** Federica Blando, Helge Berland, Gabriele Maiorano, Miriana Durante, Andrea Mazzucato, Maurizio E. Picarella, Isabella Nicoletti, Carmela Gerardi, Giovanni Mita, Øyvind M. Andersen

**Affiliations:** ^1^Institute of Sciences of Food Production, (ISPA), CNR, Lecce, Italy; ^2^Department of Chemistry, University of Bergen, Bergen, Norway; ^3^Department of Agriculture and Forest Sciences, University of Tuscia, Viterbo, Italy; ^4^Institute for Biological Systems (ISB), CNR, Rome, Italy

**Keywords:** tomato, tomato breeding, bioactive compounds, anthocyanins, functional food

## Abstract

Tomato (*Solanum lycopersicum* L.) is one of the most cultivated vegetable in the world and it represents a large source of bioactive compounds, including carotenoids and polyphenols (phenolic acids and flavonoids). However, the concentration of flavonoids in tomato is considered sub-optimal, particularly because anthocyanins are not generally present. Therefore, this crop has been the object of an intense metabolic engineering in order to obtain anthocyanin-enriched tomatoes by using either breeding or transgenic strategies. Some wild tomato species, such as *S. chilense* and *S. cheesmaniae*, biosynthesize anthocyanins in the fruit sub-epidermal tissue, and some alleles from those genotypes have been introgressed into a new developed purple tomato line, called “Sun Black” (SB). It is a tomato line with a purple skin color, both in green and in red fruit stages, due to the biosynthesis of anthocyanins in the peel, and a normal red color pulp, with a taste just like a traditional tomato. SB is the result of a breeding programme and it is not a genetically modified (GM) product. We report the chemical characterization and structure elucidation of the attractive anthocyanins found in the peel of SB tomato, as well as other bioactive compounds (carotenoids, polyphenols, vitamin C) of the whole fruit. Using one- and two-dimensional NMR experiments, the two main anthocyanins were identified to be petunidin 3-*O*-[6″-*O*-(4^‴^-*O*-*E*-*p-*coumaroyl-α-rhamnopyranosyl) -β-glucopyranoside]-5-*O*-β-glucopyranoside (petanin) and malvidin 3-*O*-[6″-*O*-(4^‴^-*O*-*E*-*p-*coumaroyl-α-rhamnopyranosyl)-β-glucopyranoside]-5-*O*-β-glucopyranoside (negretein). The total anthocyanins in the whole ripe fruit was 1.2 mg/g dry weight (DW); 7.1 mg/100 g fresh weight (FW). Chlorogenic acid (the most abundant phenolic acid) was 0.6 mg/g DW; 3.7 mg/100 g FW. The main flavonol, rutin was 0.8 mg/g DW; 5 mg/100 g FW. The total carotenoid content was 211.3 μg/g DW; 1,268 μg/100 g FW. The total phenolic content was 8.6 mg/g DW; 52.2 mg/100 g FW. The vitamin C content was 37.3 mg/100 g FW. The antioxidant activities as measured by the TEAC and ORAC assays were 31.6 and 140.3 μmol TE/g DW, respectively (193 and 855.8 μmol TE/100 g FW, respectively). The results show the unique features of this new tomato genotype with nutraceutical properties.

## Introduction

The tomato (*Solanum lycopersicum* L.) is one of the most cultivated vegetables in the world, whose production was around 182 million tons (FAOSTAT)^1^ in 2017. The average annual consumption of tomato fruit is 18 kg per capita in Europe and 8 kg in the US, with regular dietary intake in fresh, cooked and processed form, depending on the local habit (1). The tomato fruit has a high nutritional value, thanks to the presence of numerous antioxidant compounds such as carotenoids, phenolic compounds, vitamin C and E. These compounds are supposed to be responsible for the beneficial effects on health, such as reduced risk of inflammatory processes, cancer and cardiovascular diseases (2–5). Since tomatoes are an important component of the Mediterranean diet, probably their consumption contributes to the decreased risk of some chronic diseases in the Mediterranean area (6). However, among phenolic compounds, one sub-class of these secondary metabolites is scarce in tomato, i.e., flavonoids, whose concentration is considered sub-optimal, particularly because anthocyanins are not generally present in the fruit. Therefore, tomato is an ideal candidate for anthocyanin enrichment due to its widespread consumption over the world, year-round.

A great variation in size, shape and color exists in the modern tomato varieties. Some cultivated varieties show dark skin color (“black” or “purple”) as a result of mutations in carotenoid biosynthesis or chlorophyll breakdown (7). The red color of the tomato fruit results from the accumulation of lycopene, the most present carotenoid in the fruit. Conventional tomato genotypes accumulate anthocyanins on vegetative tissues, but not on fruit (8). On the contrary, the fruit of different wild *Solanum* species, closely related to cultivated tomato, accumulates anthocyanins in the peel (7, 9).

Anthocyanins belonging to the flavonoid family are a group of natural pigments represented by over 700 different molecular structures responsible for the red-blue color of many fruits and vegetables (10), having strong antioxidant properties (11). Anthocyanins are of particular interest to the agri-food industry, because of their potential health benefits, as well as their unique feature to confer vibrant colors to a variety of products (12–14). Recent studies using purified anthocyanins or anthocyanin-rich extracts on *in vitro* experimental systems have shown anti-inflammatory and anti-atherosclerotic effects (15–17). *In vivo* animal experiments and human clinical trials have given reason of the efficacy and biological activities of anthocyanins in the prevention of cardio-vascular disease (CVD) and cancer [(18–20) and references therein].

Giving these premises, it is not surprising that in the last 20 years there has been an increasing interest in developing highly consumed food, such as tomato, rich in flavonoids and particularly anthocyanins. To this purpose, transgenic approaches have been applied to modify the biosynthesis of phenylpropanoids, in order to alter the flavonoid composition of the tomato fruit. The ectopic expression in tomato of specific transcription factor (TF) genes from maize (*Lc* and *C1*), lead to 60% increase of flavonol (kaempferol) content at the whole fruit level (8). Conversely, the overexpression of TFs *Del* and *Ros1* from snapdragon (*Antirrhinum majus* L.), under the fruit-specific *E8* promoter resulted in high anthocyanin accumulation throughout the fruit of Micro-Tom, a model cultivar for tomato research (21). The same approach, using TFs *Del* and *Ros1* from *A. majus*, as used by in Butelli et al. (21) was applied to an Indian commercial cultivar (22). Recently, Scarano et al. (23) reported the engineering of a new tomato line, named “Bronze,” expressing the TFs *Del* and *Ros1* from *A. majus* (inducing anthocyanin biosynthesis), *MYB12* from *A. thaliana* (regulating flavonol biosynthesis), and the biosynthetic gene *StSy* (from *V. vinifera*) controlling the production of resveratrol.

A strategy more likely to receive consumer acceptance is the conventional breeding using interspecific crosses with wild *Solanum* species, which transfers to cultivated varieties the ability to produce anthocyanins in the fruit. Some wild tomato species, such as *S. chilense, S. cheesmaniae, S. lycopersicoides*, and *S. habrochaites*, biosynthesize anthocyanins in the sub-epidermal tissue of the fruit, and some alleles from those genotypes have been introgressed into cultivated genetic backgrounds. At Tuscia University (Viterbo, Italy), a 20-years breeding activity has produced new tomato lines by intervarietal crossing or backcrossing mutant genes (for fruit color) to commercial cultivars used as recurrent parents (24). In particular, the combination of different alleles, responsible for the anthocyanin biosynthesis (*Aft* and *atv*), led to select a line (*AftAft/atvatv*) with a “purple” skin color, due to the biosynthesis of anthocyanins in the peel, having still a red colored flesh and a taste just like a traditional tomato (25). This selection was named “Sun Black” due to the requirement of light for anthocyanin accumulation, as phytochromes are known to be involved in light-mediated regulation of anthocyanins biosynthesis (26). The molecular nature of the introgressions underlying the “Sun Black” phenotype has been recently clarified. A dominant gene located on Chr10 and encoding an *R2R3-MYB* TF activating anthocyanin biosynthesis is thought to underlie the *Aft* variant (27), although it is still uncertain which of the three paralogs located in the involved genomic region is the actual responsible for the phenotype (28). More recently, the identity of the *atv* variant has been also identified as a mutation of an *R3-MYB* located on Chr7 and acting as a repressor of anthocyanin biosynthesis (29, 30).

We report here the chemical characterization and structure elucidation of the anthocyanins found in the peel of “Sun Black” tomato, as well as nutraceutical features of the whole fruit.

## Materials and Methods

### Plant Material (Genetic Characteristics of SB Tomato)

Purple tomatoes were bred by crossing a line carrying the *Anthocyanin fruit* introgression (*Aft*, LA1996) and the *atroviolaceum* mutant line (*atv*, LA0797) as described (25).

A stable *AftAft/atvatv* purple line (line V710448, called “Sun Black,” SB) was selected in parallel with a stable line with red fruits (*aftaft/AtvAtv*) (line V710445, hereafter referred to as WT, standing for “wild type”) used as a wild type reference. The two lines were selected for having the same vegetative and fruit characteristic, except for the fruit color phenotype. The term “Sun Black” has been protected as a trademark and the cultivars selected from the Tuscia University breeding programme are now commercialized (cv “Solenero” and others). The genetic combination giving rise to anthocyanin accumulation on the tomato peel is the same exploited in other commercial genotypes, such as cv “Indigo Rose” (https://extension.oregonstate.edu/news/purple-tomato-debuts-indigo-rose). Differently from “Indigo Rose” which is an indeterminate genotype, the SB line is a semi-indeterminate genotype.

Plants of the two lines were grown in an unheated tunnel in Viterbo, Italy (42°25′07″ N, 12°06′34″ E) arranged in twin rows and grown with the same agronomic techniques and inputs, corresponding to the standard agronomic practices for fresh market tomatoes. All genotypes were grown on tutors; axillary shoots were systematically removed, and the canopy reduced to expose the fruits to natural light. Plants were left to open pollination to set fruits.

### Reagents and Standards

Reagents were purchased from various suppliers as follows: Authentic standards of kuromanin (cyanidin 3-*O*-glucoside chloride), chlorogenic acid (3-caffeoylquinic acid), gentisic acid, rutin (quercetin 3-*O*-rutinoside) (Extrasynthèse, Genay, France); the standard for carotenoids (lutein, carotene, α-carotene, β-carotene and lycopene) were purchased from CaroteNature (Lupsingen, Switzerland); gallic acid, Folin-Ciocalteu's phenol reagent, Trolox [(S)-(-)-6-hydroxy-2,5,7,8-tetramethylchroman-2-carboxylic acid], ABTS [2,2′-azino-bis (3-ethylbenzothiazoline-6-sulfonic acid)], fluorescein disodium, AAPH [2,2′-azobis (2-methyl-propionamide)], ascorbic acid, *meta*-phosphoric acid, DTT (dithiothreitol), BHT (butylated hydroxytoluene) as well as acetonitrile, ethanol, methanol, acetone, methyl tert-butyl ether, formic acid and acetic acid (all HPLC grade) (Sigma-Aldrich, St. Louis, MO, USA). In all experiments Milli-Q (Merck Millipore, Darmstadt, Germany) water was used.

### Sample Extraction

Fruits were collected from the two lines (WT and SB) at three developmental stages: mature green (MG, fruit fully developed with surface of the tomato completely green), breaker (BR, about 50% of the fruit with color changed to red) and red ripe (RR, fruit completely ripe with red color). Developmental stages in purple fruits were inferred by inspecting the blossom end that is usually lacking anthocyanin accumulation.

Tomato fruits (at least three fruits for each developmental stage) were collected from three plants of both lines, immediately cooled at 4°C, washed, cut into pieces and reduced to a fine powder with a Waring blender, in presence of liquid N_2_. The powder was then freeze-dried using a Freezone® 2.5 model 76530 lyophiliser (Labconco Corp., Kansas City, MO, USA) for 48 and stored in polyethylene tubes at −20°C (−80°C for carotenoids), until analysis. For ascorbic acid determination, aliquots of fresh fruit were stored at −80°C until analysis.

Anthocyanin extraction (for structure elucidation) was done from the peel of at least 1 kg (fresh weight, FW) of SB tomato at MG and RR stages. Freeze-dried peel (25 g) was extracted with Methanol:Water:Trifluoracetic acid (70:29.5:0.5 v/v/v), with a ratio 1/20 (W/V), o.n., in static at room temperature. After centrifugation at 3,500 g for 10 min., the extraction was repeated for 1 h in the same condition, the supernatants were combined and solvent evaporated at 32°C to 1/3 of the initial volume, then freeze-dried.

Polyphenols (including anthocyanins, for quantitative purposes) were extracted in triplicate from 100 mg freeze dried material (whole fruit), macerated overnight at 4°C in 10 mL of extraction solvent [Methanol:Ethanol:Water:Formic acid (35:35:28:2 v/v/v/v)]. After centrifugation at 3,500 g for 10 min., the extraction was repeated on a rotary shaker at room temperature, the supernatants were combined and organic solvent evaporated at 32°C, then brought to a known volume with acidified water (0.1% formic acid). Extracts were filtered through a 0.45 μm nylon membrane (PTFE) (Millipore, Bedford, MA), stored at −20°C and analyzed in triplicates within 1 month.

Carotenoids were extracted from triplicate aliquots of freeze-dried tomato powder (50 mg, whole fruit) by the method of Sadler et al. (31) modified by Perkins-Veazie et al. (32). Carotenoids were extracted with 20 mL mixture of hexane/ethanol/acetone (2/1/1 v/v/v) containing 0.05% of BHT. Samples were shaked on an orbital shaker at 180 rpm for 15 min. Then, 3 mL of distilled water was added and the suspension was centrifuged at 4,500 g for 10 min. The organic phase was dried under nitrogen, resuspended in 1 mL of ethyl acetate and analyzed by HPLC.

Ascorbic acid extraction was done from 1 g fresh tomato fruit (from freshly-grinded fruits in Waring blender with liquid N_2_) in 10 mL 5% meta-phosphoric acid, centrifuging at 10,000 g for 20 min at 4°C.

### Analysis of Anthocyanins, Other Polyphenols, Carotenoids, and Vitamin C

The concentrated filtered freeze-dried anthocyanin extract was purified using partition against ethyl acetate and Amberlite XAD-7 column chromatography. Isolation of individual compounds was performed using Sephadex LH-20 column chromatography and preparative HPLC. Structural elucidations of the main anthocyanins were mainly based on the following NMR experiments: One-dimensional ^1^H, 2D heteronuclear single quantum coherence (^1^H−^13^C HSQC), heteronuclear multiple bond correlation (^1^H−^13^C HMBC), double quantum filtered correlation (^1^H−^1^H DQF-COSY), and total correlation (^1^H−^1^H TOCSY) spectroscopy [experimental procedure according to Skaar et al. (33)]. See Table 1 for assignments of individual ^1^H and ^13^C chemical shifts for anthocyanin **1** and **2**, and Figures S1, S2 for the HSQC and HMBC spectra of anthocyanin **1**.

**Table 1:** ^1^H and ^13^C NMR spectral data for the anthocyanins petunidin 3-*O*-[6″-*O*-(4^‴^-*O*-*E*-*p-*coumaroyl-α-rhamnopyranosyl)-β-glucopyranoside]-5-*O*-β-glucopyranoside (petanin, **1**) and malvidin 3-*O*-[6″-*O*-(4^‴^-*O*-*E*-*p-*coumaroyl-α-rhamnopyranosyl)-β-glucopyranoside]-5-*O*-β-glucopyranoside (negretein, **2**) isolated from the peel of “Sun Black” tomato recorded in CF_3_COOD-CD_3_OD (5:95, v/v) at 25°C.

	**1 (^**1**^H)**	**2 (^**1**^H)**	**1 (^**13**^C)**	**2 (^**13**^C)**
**ANTHOCYANIDIN**				
2			164.34	164.66
3			146.03	146.32
4	9.06	9.02	133.92	135.41
5			156.56	156.86
6	7.11	7.04	105.63	105.90
7			169.40	169.95
8	7.16	7.14	97.24	97.76
9			156.94	157.45
10			112.82	113.31
1′			119.71	119.75
2′	8.11	8.04	109.48	111.16
3′			149.65	149.84
4′			146.35	147.35
5′			147.56	149.84
6′	7.94	8.04	113.95	111.16
OMe	4.10	4.01	57.04	57.44
**3-*****O*****-GLUCOSIDE**				
1″	5.59	5.50	102.26	106.14
2″	3.82	3.70	74.47	75.00
3″	3.67	3.59	78.29	78.58
4″	3.56	3.47	71.51	71.76
5″	3.91	3.81	77.81	78.05
6A″	4.11	4.01	67.30	67.59
6B″	3.82	3.73	67.30	67.59
**6″-*O*-RHAMNOSYL**				
1^‴^	4.80	4.71	101.63	102.13
2^‴^	3.88	3.80	72.34	72.50
3^‴^	3.92	3.82	70.34	70.61
4^‴^	5.01	4.91	75.27	75.52
5^‴^	3.83	3.71	67.17	67.71
6^‴^	1.09	0.98	17.73	18.21
**5-*****O*****-GLUCOSIDE**				
1^′*′′′*^	5.28	5.20	102.36	102.90
2^′*′′′*^	3.78	3.69	74.64	75.02
3^′*′′′*^	3.66	3.59	77.97	78.51
4^′*′′′*^	3.63	3.54	70.90	71.31
5^′*′′′*^	3.73	3.69	76.81	78.29
6A^′*′′′*^	4.03	3.94	62.05	62.37
6B^′*′′′*^	3.87	3.79	62.05	62.37
***4^‴^-E-p-*COUMAROYL**				
1			127.06	127.29
2	7.53	7.43	130.64	131.27
3	6.90	6.81	116.39	117.03
4			161.17	161.39
5	6.90	6.81	116.39	117.03
6	7.53	7.43	130.66	131.27
α	6.35	6.27	114.38	115.07
β	7.67	7.57	146.58	147.40
COO			168.80	169.06

*See Figure 2B for structures*.

Sample extracts were analyzed for phenolic compounds by an HPLC apparatus with photodiode array (PDA) detection (Agilent 1100 series, Agilent Technologies, Santa Clara, CA, USA). Instrument control, data acquisition and processing were provided by the ChemStation software (Agilent Technologies). Separations were performed at 30°C on a Luna RP-C18 column (250 × 4.6 mm, 5 μm internal diameter) (Phenomenex, Torrance, CA, USA) equipped with a guard cartridge column. The samples were eluted following the multi-segment linear gradient employed in Gerardi et al. (14), using 5% (v/v) formic acid both in water (mobile phase A) and in acetonitrile (mobile phase B). UV-visible spectra were recorded in the 200–800 nm range and chromatograms were acquired at 280 and 520 nm. Individual polyphenolic compounds were identified by comparing their peak retention times and UV-visible spectra with those of commercial standards, and by co-chromatography of samples spiked with the standards.

The identified phenolic compounds were quantified by the external standard method using calibration graphs obtained with solutions of available authentic standards at six different concentration within the linearity range of concentration.

Anthocyanins were quantified by using a calibration graph (six concentration levels from 0.001 to 0.5 μg/L, analyzed in triplicate) constructed with petanin standard purified in house.

Carotenoids analyses were carried out using an Agilent 1100 Series HPLC system as described by Durante et al. (34).

Determination of vitamin C (Ascorbic acid, AA, plus Dehydroascorbic acid, DHA) was done according to Sánchez-Moreno et al. (35), with some modifications. Samples were kept in the dark and on ice all the time. The total AA + DHA was obtained after reduction of DHA to AA by DTT. Sample extract (0.2 mL) and DTT solution (50 mg/mL) (0.2 mL) were mixed and diluted to 1 mL with 5% meta-phosphoric acid and let to react at room temperature in darkness for 2 h, then injected onto the HPLC system, following the same conditions as in Gerardi et al. (14). DHA content was obtained by subtracting the initial AA content to the final total AA content after reduction.

### Antioxidant Capacity Analysis

The Folin-Ciocalteu (F-C) reducing capacity assay, the TEAC and the ORAC assays were evaluated in WT and SB hydrophilic extracts, as described in Gerardi et al. (14). A rapid microplate methodology, using a microplate reader (Infinite M200, Tecan Trading AG, Switzerland) and 96-well plates (Costar, 96-well black round bottom plate, Corning) were used. All experiments were performed in triplicate, and two independent assays were performed for each sample.

### Statistical Analysis

Assays were carried out in triplicate and the results were expressed as mean values ± standard deviation (SD). Differences between samples were analyzed by one-way analysis of variance (ANOVA) with Tukey's HSD *post hoc* test. A *p*-value lower than 0.05 was considered statistically significant. Statistical analysis was performed using SigmaPlot version 13.0 software (SyStat Software Inc., Chicago, IL).

## Results

### Occurrence and Profile of Polyphenolic Compounds: Comparison of “Sun Black” and Wild Type Tomatoes

#### Anthocyanins

**Figure 1:**
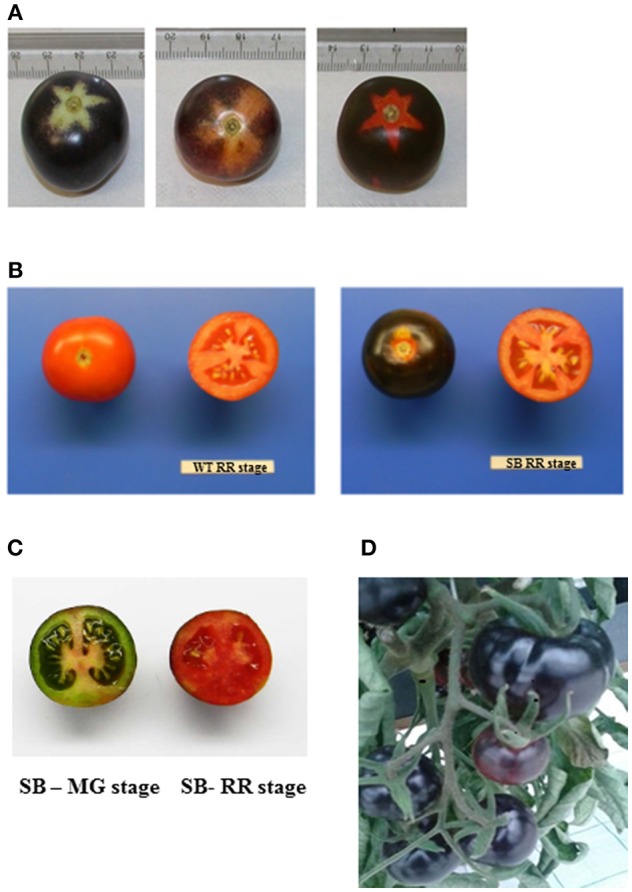
Sun Black (SB) tomato fruit. (**A**) SB at three developmental stages—mature green (MG), breaker (BR), and red ripe (RR) (from left to right). (**B**) Whole and cross section of wild type (WT) (left) and SB (right) both at RR stage. (**C**) Cross section of SB (MG, left, RR right); (D): SB tomato fruits on the vine, at RR stage.

The presence of anthocyanin pigment in SB tomato fruit was visually detected already at the immature (MG) stage, making difficult the inspection of developmental stages, unless to focus on the calix imprint or shaded area where anthocyanin biosynthesis was not activated by the light (Figure 1).

The only but very significant difference between WT and SB tomato fruits was the color of the peel (Figure 1A), instead the flesh was regularly red, both at immature (MG) and mature (RR) stage (Figures 1B,C).

**Figure 2:**
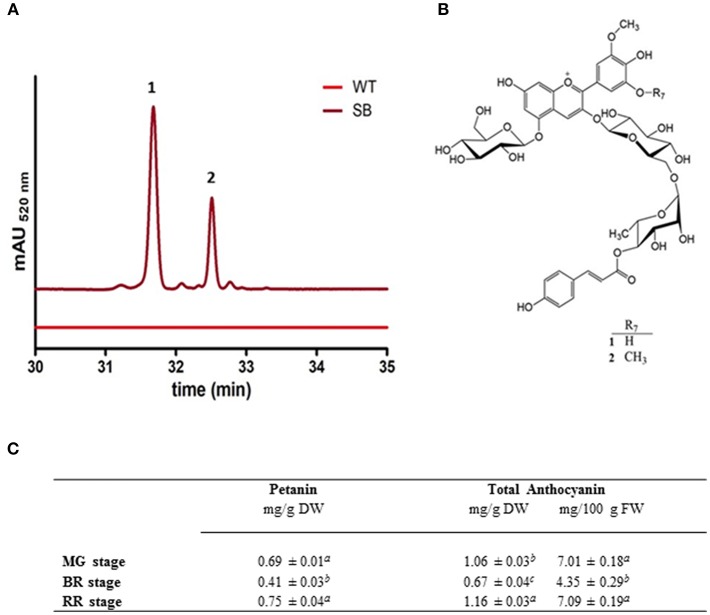
Anthocyanin characterization of SB tomato fruit extract. (**A**) Chromatografic profile of SB tomato fruit RR stage (at MG and BR stages the chromatographic profiles were similar) (**B**) structures of the main anthocyanins (petanin, **1**, and negretein, **2**) found in SB tomato peel. **(C)** Anthocyanin quantification in whole berry of SB tomato line, both as total or individual petanin.

The HPLC profile of SB fruit extracts, regardless of the ripening stage, showed two major peaks (corresponding to pigments **1** and **2**), in addition to small amounts of other anthocyanins (Figure 2A).

The downfield part of the ^1^H NMR spectrum of anthocyanin **1** showed a 1H singlet at δ 9.06 (H-4), a 2H AX system at δ 8.11 (H-2′) and δ 7.94 (H-6′), and a 2H AX system at δ 7.16 (H-8) and δ 7.11 (H-6) (Table 1). A singlet at δ 4.10 integrating for 3H, corresponding to a methoxy group, identified the aglycone as petunidin. The acyl moiety was identified as *p*-coumaric acid by the 4H AA'XX' spin system at δ 7.53 (H-2,6) and δ 6.90 (H-3,5) and the AX system at δ 7.67 (H-β) and δ 6.35 (H-α). A coupling constant of 15.8 Hz between H-α and H-β showed the *E*-configuration. The chemical shifts of the carbons of the aglycone and acyl moiety assigned by the HMBC and HSQC NMR experiments were also in agreement with the presence of petunidin and one p-coumaroyl acid moiety (Table 1). In the ^1^H NMR spectrum three anomeric proton signals were detected. The proton and carbon resonances belonging to the respective sugar moieties were assigned by DQF-COSY, TOCSY, and HSQC experiments to be in accordance with two glucopyranosyl and one rhamnopyranosyl units (Table 1).

The connection sites of the sugars of **1** on the aglycone were derived from the HMBC experiment. Cross-peaks between the anomeric proton at δ 5.59 and the carbon at δ 146.03 (C-3), and the anomeric proton at δ 5.28 and the carbon at δ 156.56 (C-5), revealed that the aglycone 3- and 5-positions were both connected to a glucopyranosyl with β-linkages showing coupling constants of 7.8 Hz. A cross-peak between the anomeric proton at δ 4.80 and the carbon at δ 67.30 revealed that the rhamnopyranosyl was connected to the 6″-position on the 3-glucopyranoside. The downfield shift (5.25 ppm) of the 6″-carbon resonance compared to the analogous signal of the 5-glucopyranosyl (Table 1) also confirmed this connection site. A downfield shift of H-4^‴^ (δ 5.01) together with a cross-peak in the HMBC spectrum between this resonance and the carbon signal at δ 168.80, revealed that the p-coumaroyl moiety was connected to the rhamnosyl 4^‴^-position. Thus, **1** was identified as petunidin 3-*O*-[6″-*O*-(4^‴^-*O*-*E*-*p-*coumaroyl-α-rhamnopyranosyl)-β-glucopyranoside]-5-*O*-β-glucopyranoside called petanin [Figure 2B; (36)].

The NMR spectra of anthocyanin **2** showed many similarities with the corresponding spectra of **1** (Table 1). After assignments of the proton and carbon resonances, pigment **2** revealed an anthocyanidin B-ring with one hydroxyl group replaced with an methoxy group compared to that of **1**, in accordance with malvidin. Thus, **2** was identified as malvidin 3-*O*-[6″-*O*-(4^‴^-*O*-*E*-*p-*coumaroyl-α-rhamnopyranosyl)-β-glucopyranoside]-5-*O*-β-glucopyranoside called negretein [Figure 2B; (37)].

Petanin and negretein represented 56.6 and 21.4% of the total anthocyanins in SB peel, respectively.

No anthocyanin compound was detected in WT tomato fruit (flesh or peel) nor in the flesh of SB fruit (data not shown). The quantification of anthocyanin in SB (whole fruit) was done using standard curves based on petanin, the standard purified in house. This has made the quantification more reliable than in other reports, where aglycone standard curves were used. Figure 2C reports the anthocyanin quantification in whole berry of SB line, both as total or individual petanin.

#### Phenolic Acids and Flavonols

For the analysis of polyphenols we used a reproducible extraction protocol using aqueous acidified methanol/ethanol as extraction solvent. This solvent was able to extract polar and semi-polar compounds, and to stabilize anthocyanins on their flavylium forms due to the acid content. Each sample was extracted in triplicate and two technical replicates were adopted to assure reliable results as recovery and repeatability.

We decided to conduct the study with the whole fruit in order to simulate the conditions while eating the fresh fruit.

**Figure 3:**
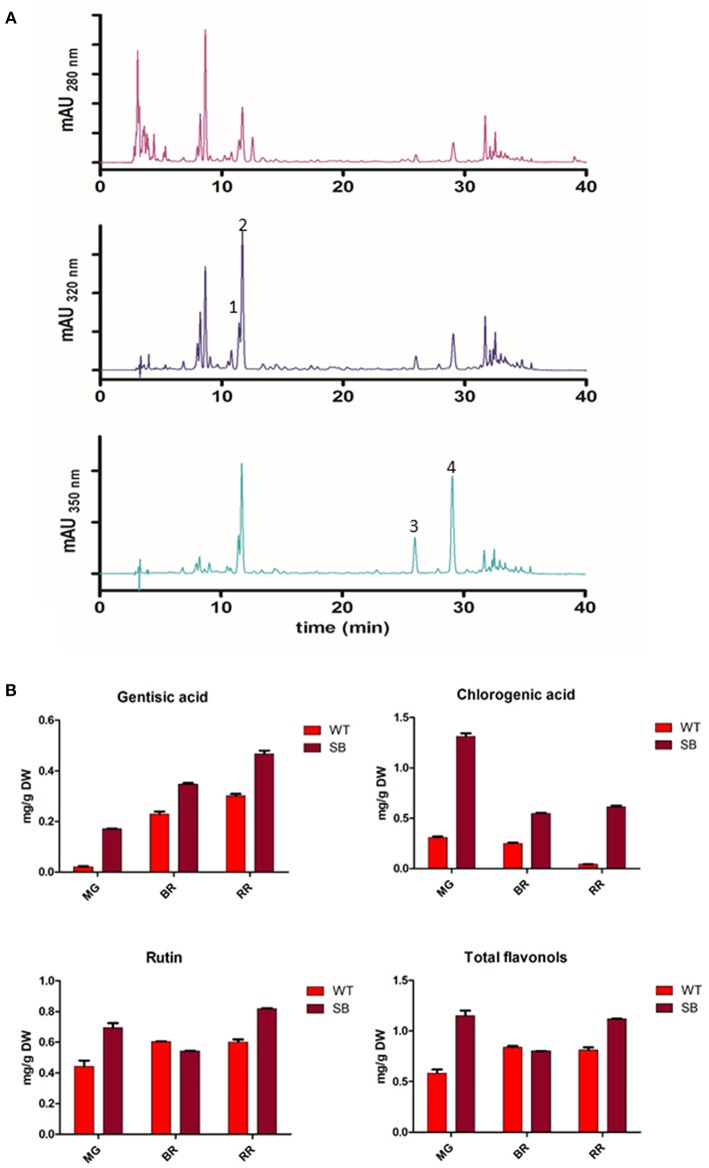
Phenolic acids and flavonols in SB tomato extract. (**A**) Chromatographic profile of SB tomato fruit extract at λ = 280-320-350 nm. (**B**) Quantification of some phenolic acids and flavonols. Peak 1, gentisic acid; peak 2, chlorogenic acid; peak 3, rutin pentoside; peak 4, rutin.

With the chromatographic method already established in our laboratory (14) we were able to identify several peaks corresponding to phenolic compounds commonly found in traditional tomatoes (38) (Figure 3A).

The chromatographic profile of SB tomato extract at 280 and 320 nm appeared much more complex than other purple tomato genotype (ex. V118 from Ontario) (39) for the more polar compounds eluting earlier (hydroxycinnamic acids). SB extract showed a group of predominant peaks, eluting before chlorogenic acid, which could be assigned, on the basis of similarity to V118 purple tomato, to the unknown peak 1, tentatively identified as caffeic acid hexoside by the authors (39).

The quantification of polyphenolic compounds is reported in Figure 3B and Table S1. In addition to anthocyanins, not present in WT, also phenolic acids and flavonols were found at higher level in SB tomato.

As a quantity, chlorogenic acid was the most abundant phenolic compound quantified in SB tomato extract (from 0.5 to 1.3 mg/g DW, depending on the developmental stage).

Among flavonols, only two peaks were present with maximum absorbance at λ = 350 nm. Rutin (RT = 29 min) was identified and quantified (Table S1). The peak with RT = 25.9 min showed an absorption spectrum similar to rutin, and from literature data on tomato, particularly in tomato bearing *high pigment* (*hp*) gene, it has been tentatively assigned to rutin pentoside (24, 38). Total flavonols were quantified as Rutin Equivalent (RE) based on the area of rutin plus rutin pentoside (Table S1).

A more detailed characterization of unknown phenolic compounds present in SB extract will be addressed in a future study; here we report a general characterization of nutraceutical components of the new bred purple tomato SB, in respect to the wild type fruit.

### Carotenoids Profile and Their Occurrence

The identification and quantification of carotenoids by HPLC is shown in Table 2.

**Table 2:** Carotenoid contents (μg/g dry weight, DW, and μg/g fresh weight, FW) in wild type (WT) and Sun Black (SB) tomato at different stages of ripening, mature green (MG), breaker (BR), and red ripe (RR).

	**Lutein**		**β-carotene**		**α-carotene**		**Lycopene**		***Total carotenoids***	
**Sample**	**μg/g DW**	**μg /100 g FW**	**μg/g DW**	**μg /100 g FW**	**μg/g DW**	**μg /100 g FW**	**μg/g DW**	**μg /100 g FW**	**μg/g DW**	**μg/100 g FW**
*WT*-MG	39.75 ± 3.29*^*a, b*^*	278.25 ± 23.03*^*a*^*	21.33 ± 2.61*^*b*^*	149.31 ± 18.27*^*b*^*	3.49 ± 0.53*^*b*^*	24.43 ± 3.71*^*b*^*	ND	ND	64.58 ± 6.44*^*c*^*	452.06 ± 45.08*^*d*^*
*WT*-BR	29.70 ± 0.58*^*c*^*	187.11 ± 4.06*^*b*^*	37.20 ± 0.86*^*b*^*	234.36 ± 5.41*^*b*^*	4.07 ± 0.61*^*b*^*	25.64 ± 3.84*^*b*^*	24.67 ± 0.84*^*c*^*	155.42 ± 5.29*^*c*^*	95.65 ± 2.9*^*b, c*^*	602.59 ±18.27*^*b, d*^*
*WT*-RR	35.58 ± 1.20*^*b, d*^*	273.96 ± 9.24*^*a*^*	46.33 ± 0.82*^*b*^*	356.74 ± 6.31*^*b*^*	4.11 ± 0.12*^*b*^*	31.64 ± 0.92*^*b*^*	75.42 ± 2.58*^*a*^*	580.73 ± 19.86*^*a*^*	161.46 ± 4.73*^*a*^*	1243.24 ± 36.42*^*a, c*^*
*SB-*MG	41.92 ± 2.55*^*a*^*	276.67 ± 16.83*^*a*^*	24.20 ± 3.88*^*b*^*	159.72 ± 25.60*^*b*^*	4.58 ± 0.15*^*b*^*	30.22 ± 0.99*^*b*^*	ND	ND	70.71 ± 6.69*^*b, c*^*	466.68 ± 44.15*^*b, d*^*
*SB*-BR	44.31 ± 1.79*^*a*^*	288.01± 11.63*^*a*^*	49.23 ± 5.34*^*b*^*	319.99 ± 34.71*^*b*^*	9.82 ± 1.49*^*a*^*	63.83 ± 9.68*^*a*^*	22.26 ± 3.58*^*c*^*	144.69 ± 23.27*^*c*^*	125.64 ± 12.22*^*b*^*	816.66 ± 79.43*^*b, c*^*
*SB*-RR	31.75 ± 2.28*^*c, d*^*	190.5 ± 13.68*^*b*^*	112.79 ± 43.11*^*a*^*	676.74 ± 258.66*^*a*^*	4.71 ± 0.25*^*b*^*	28.26 ± 1.5*^*b*^*	62.07 ± 6.0*^*b*^*	372.42 ± 36.0*^*b*^*	211.33 ± 51.65*^*a*^*	1267.98 ± 309.9*^*a*^*

*The same letters in the same column indicate that mean values (n = 3) are not significantly different (p < 0.05)*.

On a dry weight basis, at the MG stage both in WT and SB the main carotenoid was lutein, followed by β- and α-carotene, respectively, in both genotypes, while lycopene was not detected. When compared, the various carotenoids occurred at the MG stage in similar amounts in both genotypes. At the BR stage, differences could be found between WT and SB: The content of lutein and α-carotene was higher in SB than in WT. At the RR stage, the total carotenoids content was statistically similar in the SB and WT lines. However, the β-carotene content was significant higher in the SB sample, while the lycopene content was lower.

### Phenolic Content and Antioxidant Capacity

The antioxidant capacity of SB was evaluated by considering only the hydrophilic extract, which is the main contributor to the total antioxidant capacity in fruits and vegetables, particularly in the purple tomato (39).

The total phenols content was measured by the Folin-Ciocalteu (F-C) assay, as reported in Gerardi et al. (14), and it increased during ripening, in both tomato genotypes.

In case of SB tomato at MG stage, the phenolic content (5.8 mg GAE/g DW) was higher than WT by 152%, and at RR stage (8.6 mg GAE/g DW) by 134%. On a fresh weight base, at MG stage, SB had more phenols than WT by 137%, at RR stage SB had 85% more phenols than WT (Table 3).

**Table 3:** Total phenols and antioxidant capacity (by TEAC and ORAC assays) of wild type (WT) and “Sun Black” (SB) tomato at different stages of ripening, mature green (MG), breaker (BR), and red ripe (RR).

**Sample**	**Total phenolics^§^**		**TEAC^°^**		**ORAC^°^**	
	**mg GAE/g DW**	**mg GAE/100 g FW**	**μmol TE/g DW**	**μmol TE/100 g FW**	**μmol TE/g DW**	**μmol TE/100 g FW**
*WT-* MG	2.28 ± 0.26*^*d*^*	15.96 ± 1.82*^*d*^*	6.93 ± 1.21*^*d*^*	48.51 ± 8.47*^*d*^*	60.04 ± 3.24*^*c*^*	420.28 ± 23.32*^*c*^*
*WT-* BR	4.34 ± 0.34*^*c*^*	27.34 ± 2.14*^*c*^*	12.11 ± 0.92*^*c*^*	76.29 ± 5.79*^*c*^*	64.48 ± 5.80*^*c*^*	406.27 ± 36.54*^*c*^*
*WT-* RR	3.66 ± 0.37*^*c*^*	28.18 ± 2.84^c^	10.35 ± 0.82*^*c*^*	79.69 ± 6.31*^*c*^*	75.49 ± 5.93*^*c*^*	581.32 ± 45.66*^*b*^*
*SB-* MG	5.76 ± 0.35*^*b*^*	38.01 ± 2.31*^*b*^*	22.95 ± 2.53*^*b*^*	151.47 ± 16.69*^*b*^*	104.13 ± 3.05*^*b*^*	687.25 ± 20.13*^*b*^*
*SB-* BR	6.03 ± 0.41*^*b*^*	39.19 ± 2.66*^*b*^*	19.9 ± 2.08*^*b*^*	129.35 ± 13.52*^*b*^*	129.44 ± 9.28*^*a*^*	841.36 ± 60.32*^*a*^*
*SB-* RR	8.56 ± 0.08*^*a*^*	52.21 ± 0.48*^*a*^*	31.64 ± 3.91*^*a*^*	193 ± 23.85*^*a*^*	140.30 ± 9.18*^*a*^*	855.83 ± 55.98*^*a*^*

§ Total Phenols as GAE = Gallic Acid Equivalent;

° *Antioxidant Capacity as TE = Trolox Equivalent. The same letters in the same column indicate that mean values (n = 3) are not significantly different (p < 0.05)*.

The TEAC value for SB at RR stage (31.6 μmol TE/g DW) was 200% more than WT (10.3 μmol TE/g DW); instead the ORAC value for SB, at the same stage of ripening (140.3 μmol TE/g DW) was 86% higher than WT (75.5 μmol TE/g DW) (Table 3).

The antioxidant capacity of SB assessed by TEAC and ORAC at MG stage (22.9 and 104.1 μmol/TE g DW, respectively) was much lower than at RR stage, probably because of the great increase in polyphenols accumulation during ripening (from 5.8 to 8.6 mg GAE/g DW, in MG and RR, respectively).

The HPLC ascorbate determination was done on WT and SB extracts only at RR stage, revealing a much higher total ascorbic acid (AA + DHA) content in SB than WT (37.3 ± 1.4 vs. 27.1 ± 1.1 mg/100 g FW, respectively). The ascorbic acid (AA, reduced form) was 82% of total AA (30.8 ± 1.1 mg/100 g FW) in SB, while in WT was much lower (around 50%, 13.2 ± 0.79 mg/100 g FW) (data not shown).

## Discussion

Despite the remarkable success in increasing flavonoid content in tomato fruits by transgenic approaches (8, 21, 23), in the last 15 years there has been a growing interest in breeding a high flavonoid GM-free tomato (40). This interest is motivated by customers' reluctance to consume transgenic food and by restriction in EU Countries to cultivate genetically modified plants (Directive EU 2015/412).

The exploitation of the biodiversity available in the wild germplasm can allow a remarkable success for specifically exploring metabolic pathways to produce healthier food. This approach has been followed by the Soressi's group in order to combine different alleles from tomato-related wild species into cultivated tomato, promoting anthocyanin production (25, 41).

The characterization of phytochemicals in naturally bred purple tomatoes has already been reported in different genotypes, from the US accession LA1996 (9) and the Canadian purple tomato line V118 (39) to the Brasilian (42) and the Japanese ones (43). Here we report the characterization of the anthocyanin content and other nutraceuticals of “Sun Black″ (SB), an Italian tomato line with purple skin color, both in green and in red fruit stages. The latter is caused by the biosynthesis of anthocyanins in the peel, whereas the flesh is red color, similar to the wild type.

In SB tomato, the anthocyanidin (aglycone) composition has previously been reported as detected mass (*m/z*) of delphinidin, petunidin and malvidin (44). In the present study, the structure of the major anthocyanins has been elucidated for the first time.

The anthocyanin composition of SB peel is similar to the one previously reported in other purple breeding lines (9, 39). Probably all these genotypes share a common genetic base. In the V118 line (39), petunidin-derivatives accounted for approximately 91.9% of the total anthocyanins, more than in SB (which was 56.6%). In the triple mutant genotype *Aft*/*atv*/*hp2*, mostly petunidin and lower amount of delphinidin, in acylated form, were found (42).

The GM purple tomato from C. Martin's Lab. contains (both in the peel and in the flesh) acylated derivatives of delphinidin and petunidin, and at lower extent, malvidin (45). Moreover, in a recent study on a transgenic *Del/Ros1* tomato, two malvidin-based anthocyanins were reported (46).

The similarity of the anthocyanin structures (delphinidin-based) in cross-bred and transgenic tomatoes (overexpressing the *SlANT* gene) demonstrated that the biosynthetic machinery for anthocyanin biosynthesis is under control of the same *MYB* transcription factors (47), although two candidates remain to underlie the *Aft* variant (*SlANT* and *SlAN2*) (28). In addition, the first anthocyanidin product (delphinidin) is promptly methylated by the action of methyltransferases (48) in the two systems.

The anthocyanin content of SB tomato fruit (1.2 mg/g DW or 7.1 mg/100 FW) is comparable to some anthocyanin-rich vegetables (eggplant or red lettuce) or fruits (light-colored strawberries or cherries).

The not linear anthocyanin accumulation in SB tomato at different ripening stages (Figure 2C) could be explained by the different developmental behavior of the fruit: while at immature stage the biosynthesis of anthocyanin is already promptly activated (by the light), at the breaker stage the fruit undergoes cells division and expansion, with fruit enlargement and metabolite biosynthesis slowdown. At ripe stage the full development of the fruit and the anthocyanin biosynthesis (as well all the other typical metabolites of the ripe fruit) is completed.

The anthocyanin content in the peel of SB tomato is controlled by environmental factors, light in particular. It is known that the anthocyanin biosynthesis is subjected to the environmental activation on regulatory genes (*MYB* transcription factors), by light or cold (26, 49). In the environmental condition described here (unheated tunnel, Central Italy), the anthocyanin content of SB fruits was 1.16 mg/g DW. When SB tomatoes were hydroponically grown in South Italy (where light irradiation is generally higher), preliminary data suggest that at the RR stage both anthocyanin content and ORAC antioxidant capacity were 20% higher than the values reported here (data not reported). In purple tomato V118, the authors reported a total anthocyanin content of nearly 0.25 mg/g DW (39). SB tomato contains four times more anthocyanin as determined more precisely using a real standard (petanin) purified in house.

In transgenic anthocyanin-rich tomatoes a much higher anthocyanin content was reported, as expected, because of the presence of anthocyanins in the flesh (21, 46).

For colorless phenolic compounds, the HPLC chromatographic profile of the SB tomato extract was not different (for major peaks) from the WT. However, the peaks area was greater, revealing a higher content of hydrophilic polyphenolic compounds. Indeed the phenolic content assessed by Folin- Ciocalteu was higher by 100% and more in SB extract than in WT.

Gentisic and chlorogenic acids were present in SB tomato extracts at higher level in respect to WT, for each stages of ripening. Rutin was more abundant in SB, except for breaker stage. Total flavonols were not different in SB and WT at RR stage. It is interesting to note that, while during ripening the content of gentisic and chlorogenic acids followed a linear trend (increasing for gentisic, decreasing for chlorogenic), for rutin, total flavonols, and total anthocyanins the trend was not linear. As already discussed above in the anthocyanin section, during ripening and particularly at the breaker stage, the fruit undergoes many physiological and metabolic changes, not consistent with the other stages. While analyzing three stages of ripening, we focused our interest to the ripe stage, which is important for consumers.

Gentisic acid content was present in SB tomato at higher level than reported in the V118 purple tomato genotype, while chlorogenic acid was at the same level (39).

The total carotenoids content in SB (RR stage) was similar to the average amount reported for tomatoes (50), particularly for greenhouse-grown tomatoes (1), and even similar to the reported values in other purple genotypes, as the breeding line V118 from Ontario, although the carotenoid profile was different (39). Borghesi et al. (44) reported a higher content of carotenoids in SB tomatoes, and a very different pattern in lycopene, β-carotene and lutein than reported in our study on the same tomato line. In that study, tomatoes were hydroponically grown, that probably influenced the carotenoid biosynthesis. Moreover, difference in carotenoid analysis methodology can greatly influence the final metabolite quantification.

As far as the cross-talk between lipophilic carotenoids and hydrophilic flavonoids was concerned, interestingly, when transgenic lines with altered carotenoid contents were analyzed, no significant differences were found in phenolic or flavonoid content (51). Breeding or transgenic lines for increased phenylpropanoids did not show an increase of flavonoids at expenses of carotenoids (7, 21). In a transgenic tomato cultivar, total carotenoid and lycopene levels did not vary, in spite of a higher anthocyanin content of 13 mg/100 g FW (22). It has been supposed that both classes of compounds have different role and subcellular localization (plastids for carotenoids vs. vacuoles for anthocyanins), and do not compete for precursors (42).

In tomatoes bearing *high pigment* (*hp*) gene, with phenotypic effects similar to those due to the *Aft* locus, a slight increased level of carotenoids and polyphenols in respect to the control line was reported (24).

Recently, the potential health benefits of anthocyanins have been related to not yet identified chemical properties beyond the antioxidant capacity of the molecules (12). However, the antioxidant properties of a food extract, assessed *in vitro*, is an inceptive parameter to be tested when studying the nutraceutical properties of a food. Our results on antioxidant capacity of SB hydrophilic extract show a significantly higher value in respect to WT.

The phenolic content (by F-C assay) of SB tomato, at all stage of ripening, was significantly higher than that one found in WT, and in other selected tomato lines (24). At RR stage of ripening phenolic content of SB tomato was higher than the reported value for the Canadian purple tomato V118 (39).

Two different chemical assays (TEAC and ORAC) have been adopted for evaluating the antioxidant capacity of SB extract, as it has been recommended in order to take into account the different mechanisms of action (52). In both assays, the antioxidant capacity (hydrophilic) of SB tomato, at the three stages of ripening, was significantly higher than WT at the same stage, due to the presence of anthocyanin compounds in the peel, and to an increased content of other polyphenolic compounds.

The TEAC of WT tomato was in agreement with the reported value for commercial tomato cultivar (1, 53). The TEAC value for SB tomato was 200% more than WT, thanks to the increased polyphenolic content. The ABTS assay has been previously applied to SB tomato samples, on skin area with different level of pigmentation, revealing increased antioxidant capacity with increasing anthocyanin content, although not comparable to our results which are on whole fruit (54). The TEAC antioxidant capacity of our SB tomato was lower, as expected, than the value reported for transgenic tomatoes, because of the anthocyanin presence at whole berry fruit (21).

The antioxidant capacity (as ORAC assay) in SB tomato, at all stage of ripening, was statistically higher than that one found in WT. At RR stage of ripening, ORAC was lower in SB than in the Canadian purple tomato V118, in spite of a higher phenolic content in SB (39).

Tomato fruit is considered a good source of vitamin C. The vitamin C content was quite high both in WT and SB, with reference to commercial cultivar or purple tomato (1, 42). SB extracts at RR stage had a higher vitamin C content than WT. The ascorbic acid (AA, reduced form) was much higher in SB than WT. One possible explanation is the protective function of the increased polyphenol content in SB, which protects AA from oxidation. Anyway, the contribution of the ascorbic acid to the antioxidant capacity of plant extract is usually low. As it has been reported, the main contributor to the antioxidant capacity of hydrophilic extracts is not ascorbate but rather polyphenols (55).

It has been reported that purple tomatoes both obtained by transgenesis or conventional breeding showed improved shelf life compared to wild-type, with a delayed ripening and a reduced pathogen susceptibility, due to anthocyanin accumulation and increased level of natural antioxidant (54, 56). Postharvest losses by pathogenic infection can reduce the yield of vegetable crops, therefore the harvest of tomato when green and firm, and successive ethylene exposition, is a usual technique to induce color and ripeness, without developing flavor and aroma. This is likely the main reason why the marketed tomatoes are often characterized by extremely low flavor regardless of the cultivar. Preliminary panel tests on SB at commercial stage of ripeness showed a good performance for flavor, aroma and texture (data not reported). Moreover, ripening SB tomatoes showed that the ethylene climacteric peak was delayed, resulting in a higher firmness at commercial stage of ripening (57). Since SB has been bred to promote fresh consumption of a tomato rich in bioactive compounds, all this information taken together make SB tomato valuable both for nutraceutical and market qualities.

## Conclusion

From the Kuopio study which demonstrated that the consumption of large amount of anthocyanin-rich food significantly lowered the risk of CVD (58) to the recent advancement in anthocyanin health benefits (12), it can be concluded that consuming anthocyanin-rich fruits and vegetables is a recommended style of eating.

Transgenesis has been always seen in a suspicious way, due to the consumer's concerns over the consumption of GM foods. Moreover, EU rules discourage the application of transgenic cultivations. Therefore, the naturally bred SB tomato can be a valuable alternative to transgenic purple tomato, despite its lower concentration of anthocyanins and antioxidant phytochemicals. On the other hand, the anthocyanin (and phenolic) content of SB is comparable to some anthocyanin-rich vegetables (eggplant or red lettuce) or fruits (light-colored strawberries or cherries), with the advantage that tomatoes are highly consumed in many countries, and are available all year-round. In conclusion, consumption of SB tomatoes can contribute to ameliorate human health, thanks to the increased content of nutraceutical compounds.

## Data Availability

All datasets generated for this study are included in the manuscript and/or the Supplementary Files.

## Author Contributions

FB, HB, GMa, MD, IN, and CG performed the experiments. AM and MP produced the plant material. FB, IN, GMi, HB, and ØA designed the experiments. FB, HB, GMa, MD, IN, GMi, and ØA analyzed and interpreted the data. FB wrote the manuscript with the input from all the authors.

### Conflict of Interest Statement

AM and MP are participating as inventors in owing the trademark “Sun Black” and the rights on the registered variety “Solenero.” The ownership is the University of Tuscia, Viterbo, Italy. The remaining authors declare that the research was conducted in the absence of any commercial or financial relationships that could be construed as a potential conflict of interest.

## Footnotes

^1^FAOSTAT. Available online at: http://www.fao.org/faostat/en/#home
