# Supplementary material for: Nutraceutical Characterization of Anthocyanin-Rich Fruits Produced by “Sun Black” Tomato Line
Source: Front Nutr. 2019 Aug 28;6:133. doi: 10.3389/fnut.2019.00133 (PMC6722425; doi:10.3389/fnut.2019.00133)
Supplement: Supplementary file 1 [file Table_1.pdf]

TABLE S1 | Content of gentisic acid, chlorogenic acid, rutin and total flavonols in wild type (WT) and Sun Black (SB) tomato at different stages of ripening, mature green (MG), breaker (BR) and red ripe (RR).

Sample	Gentisic acid		Chlorogenic acid		Rutin		Total flavonols	
	mg/g DW	mg/100 g FW	mg/g DW	mg/100 g FW	mg/g DW	mg/100 g FW	mg/g DW	mg/100 g FW
***WT* - MG**	0.021 ± 0.003[f]	0.149 ± 0.020[e]	0.309 ± 0.013[d]	2.221 ± 0.094[c]	0.442 ± 0.054[e]	3.180 ± 0.392[b]	0.583 ± 0.052[b]	4.200 ± 0.371[c]
***WT* - BR**	0.229 ± 0.014[d]	1.445 ± 0.088[c]	0.249 ± 0.012[e]	1.571 ± 0.075[d]	0.603 ± 0.003[bd]	3.800 ± 0.019[b]	0.839 ± 0.019[b]	5.284 ± 0.121[b]
***WT* - RR**	0.301 ± 0.012[c]	2.314 ± 0.092[b]	0.043 ± 0.002[f]	0.328 ± 0.015[e]	0.602 ± 0.023[b]	4.632 ± 0.176[a]	0.811 ± 0.038[a]	6.247 ± 0.296[a]
***SB* - MG**	0.099 ± 0.005[e]	0.652 ± 0.032[d]	1.314 ± 0.041[a]	8.674 ± 0.272[a]	0.694 ± 0.069[b]	4.581 ± 0.458[a]	1.152 ± 0.069[a]	7.603 ± 0.458[a]
***SB* - BR**	0.346 ± 0.008[b]	2.251 ± 0.051[b]	0.546 ± 0.009[c]	3.551 ± 0.059[b]	0.542 ± 0.001[cde]	3.523 ± 0.004[b]	0.803 ± 0.001[b]	5.218 ± 0.004[a]
***SB* - RR**	0.467 ± 0.018[a]	2.846 ± 0.112[a]	0.613 ± 0.018[b]	3.740 ± 0.111[b]	0.818 ± 0.005[a]	4.990 ± 0.030[a]	1.119 ± 0.005[a]	6.825 ± 0.030[a]

The same letters in the same column indicate that mean values (n=3) are not significantly different ($p < 0.05$).
