# Supplementary material for: Nutraceutical Characterization of Anthocyanin-Rich Fruits Produced by “Sun Black” Tomato Line
Source: Front Nutr. 2019 Aug 28;6:133. doi: 10.3389/fnut.2019.00133 (PMC6722425; doi:10.3389/fnut.2019.00133)
Supplement: Supplementary file 2 [file Data_Sheet_1.PDF]

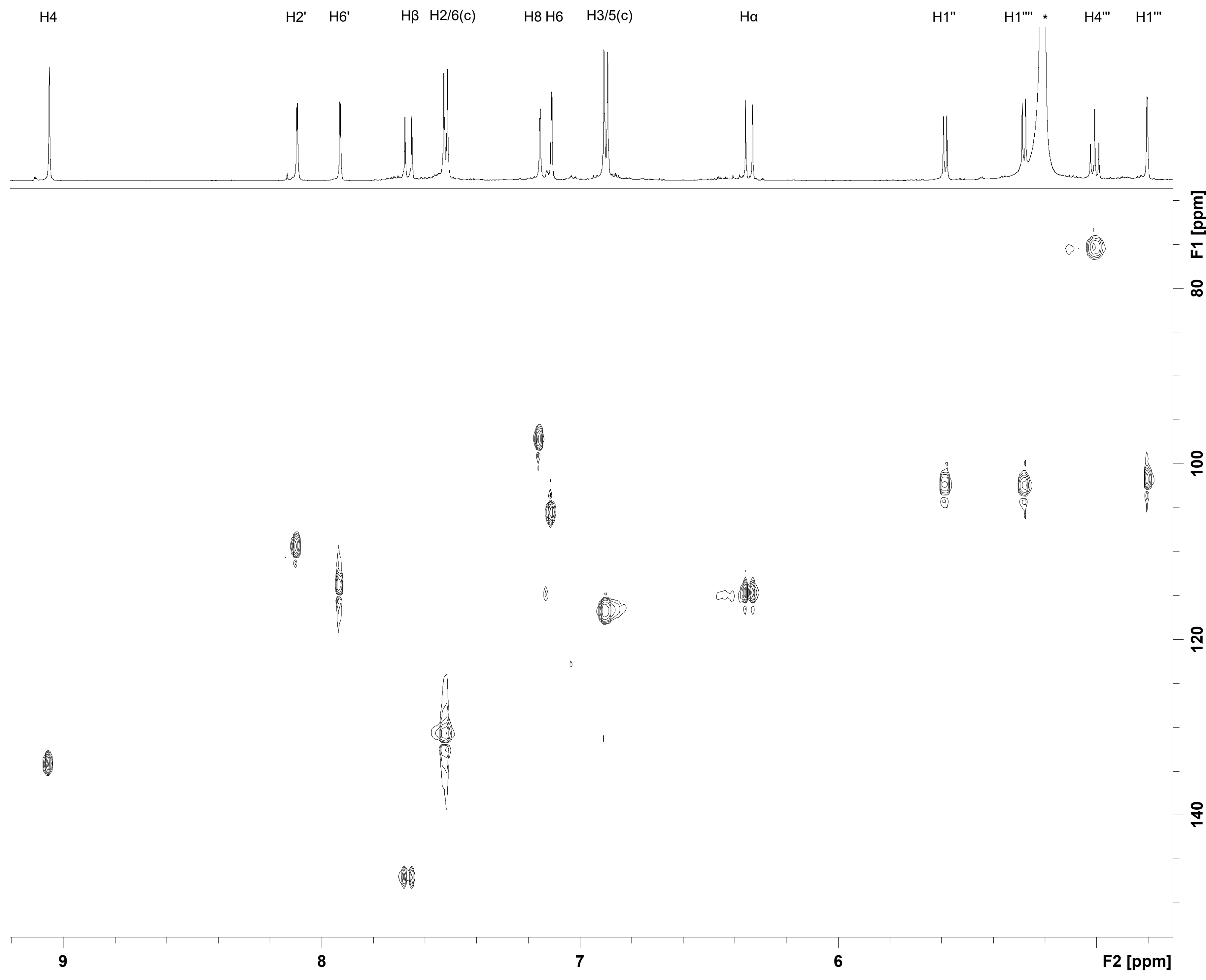

H4
H2'
H6'
Hβ
H2/6(c)
H8 H6
H3/5(c)
Hα
H1''
H1''''
*
H4'''
H1'''
F1 [ppm]
80
100
120
140
9
8
7
6
F2 [ppm]
