# Supplementary figures and images for: Nutraceutical Characterization of Anthocyanin-Rich Fruits Produced by “Sun Black” Tomato Line
Source: Front Nutr. 2019 Aug 28;6:133. doi: 10.3389/fnut.2019.00133 (PMC6722425; doi:10.3389/fnut.2019.00133)

H4 H2' H6' H $\beta$  H2/6(c) H8 H6 H3/5(c) H $\alpha$  H1'' H1''' \* H4''' H1'''

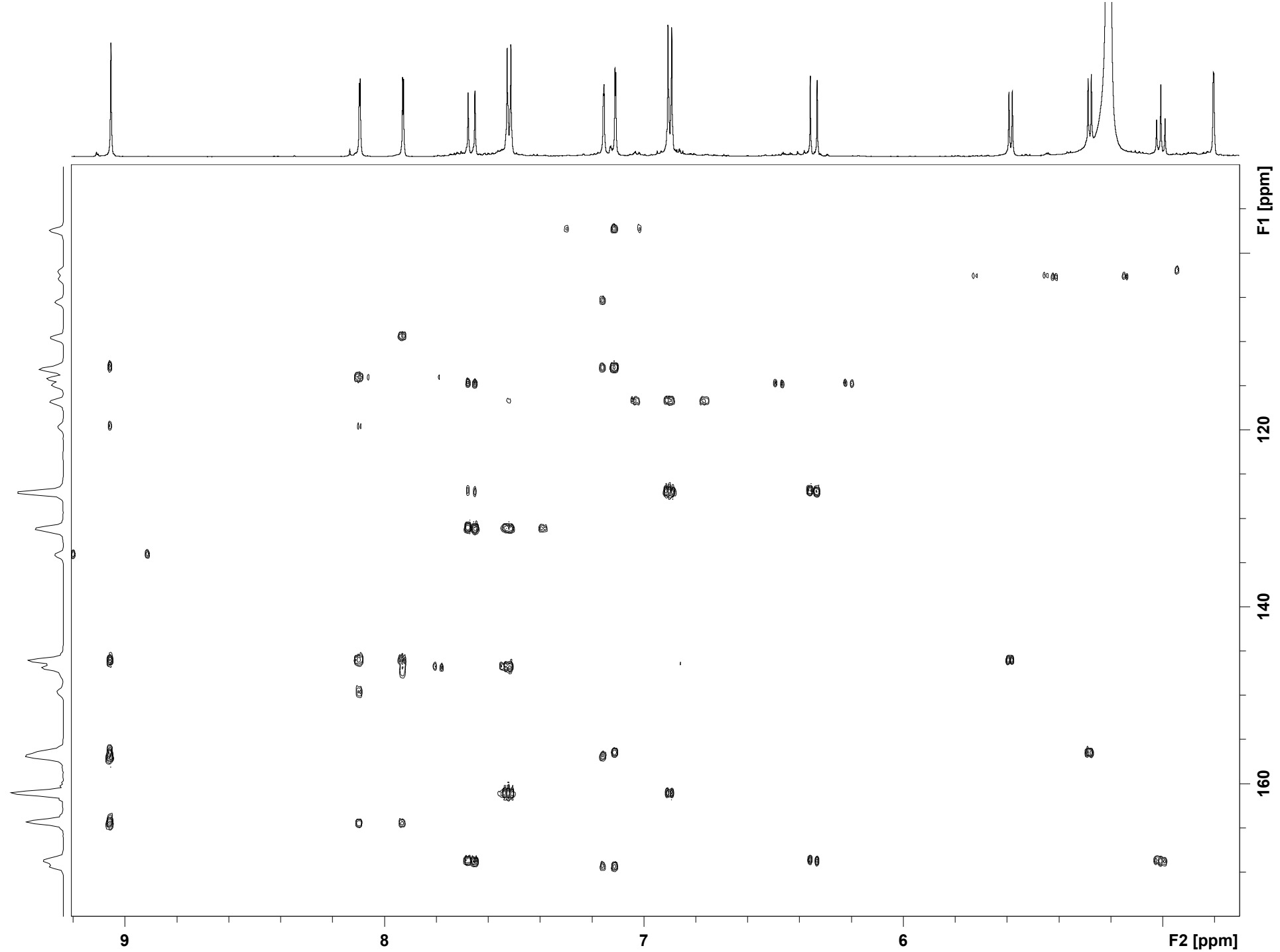

Supplement: Supplementary file 3 [file Data_Sheet_2.PDF]
