# Supplementary material for: Nutraceutical Characterization of Anthocyanin-Rich Fruits Produced by “Sun Black” Tomato Line
Source: Front Nutr. 2019 Aug 28;6:133. doi: 10.3389/fnut.2019.00133 (PMC6722425; doi:10.3389/fnut.2019.00133)
Supplement: Supplementary file 4 [file Data_Sheet_3.docx]

**Data sheet 3**

**Figure S1**: The aromatic and sugar anomeric part of the heteronuclear single quantum coherence (^1^H–^13^C HSQC) NMR spectra of anthocyanin **1**, petunidin 3-*O*-[6''-O-(4'''-*O-E-p*-coumaroyl-α-rhamnopyranosyl)-β-glucopyranoside]-5-*O*-β-glucopyranoside (petanin) isolated from the peel of ‘Sun Black’ tomato, recorded in CF3COOD-CD3OD (5:95, v/v) at 25 °C. (c) = *p*-coumaroyl moiety; * = water residual peak.

**Figure S2**: The aromatic and sugar anomeric part of the heteronuclear multiple bond quantum coherence (^1^H–^13^C HMBC) NMR spectra of anthocyanin **1**, petunidin 3-*O*-[6''-O-(4'''-*O-E-p*-coumaroyl-α-rhamnopyranosyl)-β-glucopyranoside]-5-*O*-β-glucopyranoside recorded in CF3COOD-CD3OD (5:95, v/v) at 25 °C. (c) = *p*-coumaroyl moiety; * = water residual peak.
